# Impact of a Long-Term Home-Based Rehabilitation Program on Quality of Life, Balance, and Autonomy in Adults with Disabilities

**DOI:** 10.3390/jfmk10010024

**Published:** 2025-01-07

**Authors:** Patricio Barria, Asterio Andrade, Alejandro Yelincic, Bessié Córdova, Felipe Covarrubias-Escudero, Carlos Cifuentes, Juan Pablo Appelgren-Gonzalez

**Affiliations:** 1 Research and Development Area, Corporación de Rehabilitación Club de Leones Cruz del Sur, Punta Arenas 6210005, Chile; pbarria@rehabilitamos.org (P.B.); aandrade@rehabilitamos.org (A.A.); ayelincic@rehabilitamos.org (A.Y.); bessie.cordova@rehabilitamos.org (B.C.); 2 Translational Research Unit, Trainfes Center, Santiago 8760903, Chile; felipe@trainfes.com; 3 Department of Kinesiology, Faculty of Art and Physical Education, Universidad Metropolitana de Ciencias de la Educación, Santiago 7750332, Chile; 4 Bristol Robotics Laboratory, University of the West of England, Bristol BS16 1QY, UK; carlos.cifuentes@uwe.ac.uk; 5 Center of Biomedical Imaging, Pontificia Universidad Católica de Chile, Santiago 7510000, Chile

**Keywords:** rehabilitation outcomes, functional independence, emotional well-being, therapeutic dosing, patient-centered care

## Abstract

Background: Rehabilitation is a critical process for enhancing functionality, independence, and quality of life in individuals with disabilities. Grounded in the biopsychosocial model, it addresses physical, emotional, and social dimensions through personalized, evidence-based interventions. By integrating standardized assessments and continuous evaluation, rehabilitation has the potential to promote recovery and support active participation in society. Objectives: This study evaluated the impact of a long-term, multidisciplinary, home-based rehabilitation program on quality of life, balance, and functional autonomy in adults with neuromusculoskeletal disabilities. Methods: A total of 559 participants received individualized interventions from a team of physical therapists, occupational therapists, psychologists, and other health professionals. Functional independence, balance, depressive symptoms, and quality of life were assessed using the Barthel Index, Berg Balance Scale, Beck Depression Inventory, and SF-36 questionnaire, respectively. Results: A longitudinal analysis comparing pre- and post-intervention outcomes revealed statistically significant improvements (*p* < 0.001) across all metrics. The Barthel Index median increased from 85 to 90 points, indicating greater functional independence, while the Berg Balance Scale improved from 39 to 47 points, reflecting reduced fall risk. Depressive symptoms decreased, with Beck Depression Inventory scores dropping from 12 to 9, and both physical and mental health components of the SF-36 showed marked enhancements. Conclusions: These findings demonstrate the program’s effectiveness in addressing both physical and emotional needs, emphasizing the value of extended, personalized, home-based care in improving health, autonomy, and overall quality of life for individuals with disabilities. This study underscores the potential of multidisciplinary approaches to support long-term rehabilitation in diverse populations.

## 1. Introduction

People with neuromusculoskeletal disabilities often face significant limitations in their physical, emotional, and social functionality, which negatively affects their quality of life and restricts their full participation in society [1,2]. In this context, rehabilitation programs play a crucial role in offering a path toward the recovery of independence and the improvement of patients’ functionality [3]. These programs adopt a comprehensive approach, grounded in the biopsychosocial model, encompassing physical, psychological, and social aspects. This approach aims not only to achieve physical recovery but also to fully integrate the individual into their environment, addressing their functional needs and promoting their overall well-being [4]. Rehabilitation should be integrated into healthcare systems as a continuous process parallel to medical care, enabling patients to maintain autonomy and engage in meaningful activities from the early stages of recovery, thus avoiding the loss of skills and confidence associated with prolonged inactivity [5,6].

Rehabilitation, particularly in cases of neurological disorders, is essential for restoring functionality and improving quality of life. Grounded in experience-dependent brain plasticity, this process employs strategies such as task-oriented repetitive training, multidisciplinary therapies, and home-based self-management. These techniques, tailored to the complexity and individual needs of each patient, have proven effective in promoting functional recovery and independence [7,8,9].

In this context, therapeutic dosing plays a pivotal role: achieving a minimum threshold of repetitions during exercises is fundamental to inducing plastic changes in the central nervous system [10]. Recent studies emphasize that high-intensity and frequent interventions, initiated early within the first three months post-injury, significantly enhance treatment efficacy. These interventions not only improve mobility and balance, particularly in patients with moderate complexity but also optimize clinical outcomes and support sustainable recovery [7,11,12].

The standardized and longitudinal assessment of the clinical and functional aspects of each patient is a cornerstone of the rehabilitation process. Through comprehensive evaluation, healthcare professionals can deeply understand the unique characteristics of each patient, enabling effective monitoring of progress and intervention outcomes. This insight facilitates the design of tailored therapeutic strategies that address specific needs. According to Bickenbach and Stucki (2021), rehabilitation should be viewed as a 21st-century health strategy focused on optimizing functionality and reducing disability in individuals with health conditions through a function-centered approach [13]. The implementation of this comprehensive approach, encompassing physical, emotional, and social dimensions, supports rehabilitation strategies that improve patients’ quality of life, optimize their functionality, and encourage their active participation in society [5,14].

Quality of life is a central aspect of the rehabilitation of people with disabilities, as physical and emotional well-being are fundamental components for achieving a fulfilling and satisfying life. Assessing quality of life allows healthcare professionals to identify key areas of intervention that contribute to improving both the physical functioning and psychological well-being of the patient, essential aspects of their recovery process. Among the available tools, the SF-36 questionnaire has become one of the most widely used instruments for measuring quality of life in terms of physical and emotional functionality, providing a structured framework that facilitates the design of specific and effective interventions [15].

Functional autonomy is a fundamental dimension of rehabilitation, as it enables people with disabilities to regain the ability to perform basic activities of daily living, promoting their independence and reducing the burden on their caregivers. This autonomy is essential in the rehabilitation process, as the ability to achieve functional independence has a direct impact on the patient’s social integration. The Barthel Index, a widely recognized tool for measuring this autonomy, classifies patients into different levels of dependency, facilitating the personalization of interventions according to the needs of everyone [16,17].

Similarly, emotional well-being plays a critical role in the recovery of people with disabilities, as it directly affects their motivation and willingness to actively participate in the rehabilitation process. According to Turner and Kelly (2000), patients with chronic illnesses, like many individuals with disabilities, often experience significant emotional strain due to physical and social limitations, which can lead to the onset of disorders such as depression and anxiety [18]. These symptoms affect their ability to adapt to their situation and adhere to recommended treatments. The Beck Depression Inventory (BDI) is a widely used tool for assessing the severity of depressive symptoms, enabling healthcare professionals to provide personalized interventions that promote better treatment adherence and contribute to improving the patient’s quality of life [19]. Addressing these emotional aspects in rehabilitation is essential for optimizing outcomes and fostering a more comprehensive recovery process.

Balance and the risk of falls are critical factors in rehabilitation, especially for individuals with physical disabilities or limited mobility. The Berg Balance Scale is a widely validated tool that measures balance levels and assesses fall risk in patients. Its psychometric properties have proven to be reliable and valid in both clinical and research contexts, enabling professionals to design specific interventions to improve stability and reduce the risk of accidents [20]. Furthermore, research indicates that balance impairments significantly increase the risk of falls among community-dwelling older adults, highlighting the importance of evaluating and addressing these aspects as an integral part of rehabilitation [21]. These improvements not only enhance safety but also promote patients’ independence and active participation in their daily activities.

In line with the comprehensive approach described, the present study aims to characterize the clinical and functional profile of patients by analyzing standardized variables across the dimensions of quality of life, functional autonomy, balance, and emotional state. Through a longitudinal evaluation, this study seeks to observe the effectiveness of interventions in terms of health and well-being, enabling continuous adjustments to therapeutic strategies. This study aims not only to measure progress in each of these areas but also to understand how advancements in these dimensions contribute to comprehensive functional recovery that meets the biopsychosocial needs of patients.

## 2. Materials and Methods

### 2.1. Participants

**Figure 1:**
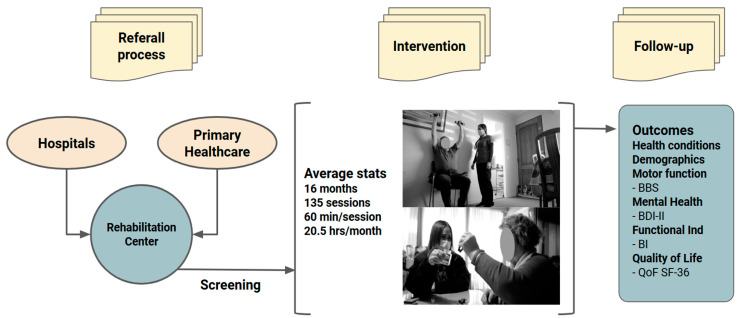
Representation of the enrollment, intervention, and principal assessments implemented in this study. BBS: Berg Balance Scale; BDI-II: Beck Depression Inventory II; BI: Barthel Index; QoF: Quality of Life.

This study included 559 patients with neuromusculoskeletal disabilities who participated in a rehabilitation program. The protocol targeted individuals registered in the database of Rehabilitation Center Club de Leones Cruz del Sur. Patients were included if they had medical referrals and clinical information available in the rehabilitation center’s database (Figure 1). This inclusion criterion ensured access to documented medical histories for accurate health status evaluation and follow-up. Exclusion criteria included individuals with severe cognitive impairment that hindered effective participation in the rehabilitation program, as well as patients with acute medical conditions requiring immediate hospitalization. More details about the program can be found in the clinical registration: Trial registration at ClinicalTrials.gov NCT06537791.

### 2.2. Intervention

The rehabilitation program was designed and implemented following a comprehensive, multidisciplinary framework tailored to the diverse needs of individuals with neuromusculoskeletal disabilities. Personalized treatment plans were systematically developed and designed by the multidisciplinary team of the Rehabilitation Center Club de Leones Cruz del Sur based on medical prescriptions obtained during baseline evaluations. These plans adhered to a patient-centered approach, grounded in the biopsychosocial model, addressing the physical, emotional, and social dimensions of rehabilitation [22]. Therapeutic goals were established using the SMART criteria (Specific, Measurable, Achievable, Relevant, and Time-bound), ensuring precision, feasibility, and alignment with the patient’s health priorities [23]. Patients were actively involved in the goal-setting process, allowing for the co-creation of meaningful objectives and activities aligned with their values and rehabilitation goals.

The intervention had a median duration of 16 months (interquartile range: 9–19 months), encompassing approximately 135 therapy sessions (interquartile range: 99–152 sessions). These sessions included physical rehabilitation, independence training through occupational therapy, and emotional and psychological support, with additional healthcare services provided by nursing staff. Each session lasted approximately 60 min, with an average of 20.5 therapy hours delivered per month. The program’s primary objectives were to enhance functional independence and improve quality of life through structured and individualized care (Figure 1).

To monitor clinical progress, two standardized medical evaluations were conducted at the beginning and end of the program. Social workers carried out detailed admission and discharge assessments to address social and environmental factors influencing the patient’s rehabilitation process. This patient-centered methodology ensured that interventions were tailored to individual needs and facilitated meaningful improvements in health, autonomy, and overall quality of life.

### 2.3. Outcome Measures

#### 2.3.1. Patient Characteristics and Health Conditions

Clinical and demographic characteristics, including age distribution, were recorded and analyzed. This study focused on evaluating changes in clinical and quality-of-life variables, such as functional independence, balance, depressive symptoms, and quality of life.

#### 2.3.2. Motor Function

Balance was assessed using the Berg Balance Scale (BBS) during the initial and final evaluations. The BBS is a validated tool that evaluates balance and fall risk in patients through 14 items measuring static and dynamic balance on a scale of from 0 to 56 points. Scores below 45 indicate a fall risk and potential need for assistance, while higher scores suggest a stable balance. The BBS has demonstrated excellent reliability, including inter-rater, intra-rater, and test–retest reliability, with intraclass correlation coefficients (ICCs) exceeding 0.90. Its validity is also well established, showing strong construct, concurrent, and predictive validity, with significant correlations to other balance and mobility assessments [24]. The BBS has proven effective in populations with neuromusculoskeletal disabilities and is widely used in rehabilitation programs [20].

#### 2.3.3. Mental Health

Mental health was assessed through depressive symptoms using the Beck Depression Inventory-II (BDI-II), a self-administered tool that measures depression severity across 21 items. BDI-II scores are classified into the following categories: minimal (0–13), mild (14–19), moderate (20–28), and severe (29–63). Changes in BDI-II scores help evaluate improvements or declines in the patient’s emotional well-being. The BDI-II has demonstrated excellent reliability, with internal consistency coefficients (Cronbach’s alpha) ranging from 0.89 to 0.91 across diverse populations. Test–retest reliability has also been established, with ICCs above 0.80 in both clinical and non-clinical samples [25]. Additionally, the BDI-II exhibits strong validity, including construct and criterion validity, as shown by its significant correlations with other depression measures, such as the Hamilton Depression Rating Scale (HDRS) and clinical diagnostic interviews [26]. The BDI-II is widely used and has demonstrated high reliability and validity for measuring depressive symptoms in clinical populations [19].

#### 2.3.4. Functional Independence

Functional independence was measured using the Barthel Index (BI), a scale that evaluates an individual’s ability to perform basic activities of daily living (ADLs) independently. The index scores range from 0 to 100, with higher scores indicating greater independence. This tool is widely used in rehabilitation to assess changes in patients’ functional capacity and categorize dependency levels, making it effective for monitoring progress in functional recovery [27]. The BI has demonstrated high reliability, with internal consistency coefficients exceeding 0.90 and test–retest reliability up to 0.95 in diverse clinical populations [28]. Furthermore, the BI exhibits strong validity, as evidenced by significant correlations with other functional measures, such as the Functional Independence Measure (FIM) [29], and its proven ability to predict meaningful clinical outcomes in populations with neuromuscular and orthopedic conditions [30].

#### 2.3.5. Quality of Life

Quality of life was assessed using the physical and mental components of the SF-36 questionnaire, a widely validated tool that measures two main components: physical and mental. This questionnaire is essential for evaluating the impact of rehabilitation interventions on patients’ daily lives. Scores range from 0 to 100, with higher values indicating better perceptions of health and emotional well-being. The SF-36 is a widely validated and commonly used tool in quality-of-life studies involving populations with disabilities and chronic illnesses [15]. The SF-36 has demonstrated high reliability, with Cronbach’s alpha values ranging from 0.70 to 0.91 across most domains and test–retest reliability coefficients ranging from 0.73 to 0.96 in various populations [31]. Its validity is well-established, showing strong correlations between the physical and mental health domains and related clinical measures. Furthermore, the SF-36 effectively differentiates between populations with varying health conditions, underscoring its utility in diverse clinical and research settings [32].

### 2.4. Statistical Analysis

The data were organized and cleaned using Microsoft Excel 365, which was employed for data preprocessing and preparation prior to statistical analysis. Descriptive and inferential statistical analyses were performed using GraphPad Prism v8.0.2 (GraphPad Software, Inc., San Diego, CA, USA), a software specifically designed for advanced scientific analysis and detailed graph generation, and PAST 4.12b, a versatile statistical tool used for non-parametric tests and specialized analyses.

Measures of central tendency, such as the median and interquartile range, were used due to the non-normal distribution of most variables. The Shapiro–Wilk test was applied to verify normality, and non-parametric tests were conducted as necessary.

The Wilcoxon signed-rank test was used to evaluate changes in paired samples (pre- and post-intervention), while the Mann–Whitney test assessed absolute changes between groups. The visualization of results included graphs and tables generated with GraphPad Prism, facilitating the interpretation of the program’s impact on clinical and quality-of-life variables.

### 2.5. Ethical Considerations

This study was approved by the Institutional Review Board of the Rehabilitation Center Club de Leones Cruz del Sur (approval code CRCS_UID_010223), ensuring compliance with ethical and methodological standards. All data were handled confidentially and anonymized. Participants did not receive any compensation for their participation in this study.

## 3. Results

### 3.1. Age

The normality analysis of the age distribution showed that this variable does not follow a normal distribution, as confirmed by the Shapiro–Wilk test (*p* < 0.001). The median age of the 559 patients was 71 years, with an interquartile range of from 59 to 81 years, indicating a predominantly older adult population across the sample.

### 3.2. Diagnosis

The frequency distribution of the primary diagnoses within the sample was as follows: osteoarthritis (25.6%), fibromyalgia (14.5%), stroke (8.23%), Parkinson’s disease (8.05%), reduced mobility and sarcopenia (8.41%), polyneuropathy (6.62%), low back pain (3.22%), amputations (2.86%), tendinopathies, fractures, and muscle injuries (7.87%), other musculoskeletal conditions (8.6%), and other neurological conditions (8.05%).

#### 3.2.1. Diseases of the Nervous System

The group of Nervous System Diseases (NSDs) comprises diagnoses primarily affecting the brain, spinal cord, and peripheral nerves, accounting for a total of 229 patients, which represents approximately 45.1% of the total sample. These conditions frequently involve impairments in motor control, sensory processing, and autonomic regulation, which are critical for maintaining functionality and independence. Common diagnoses in this group include stroke, Parkinson’s disease, polyneuropathies, and other conditions that affect the central and peripheral nervous systems. Examples include sequelae of cerebrovascular diseases, such as cerebral infarction and subarachnoid hemorrhage, as well as spinal cord injuries and diabetic polyneuropathy. Therapeutic interventions for the NSD group focus on optimizing motor function, managing chronic symptoms, and improving autonomy through evidence-based, multidisciplinary strategies. 

#### 3.2.2. Diseases of the Musculoskeletal System

The group of Musculoskeletal System Diseases (MSDs) includes 259 patients, representing 54.9% of the total sample. These conditions primarily affect bones, muscles, tendons, and joints, leading to pain, stiffness, and significant limitations in mobility and physical function. Unlike NSDs, impairments in this group are predominantly structural, such as joint degeneration, muscle atrophy, or skeletal injuries. Common diagnoses include osteoarthritis, sarcopenia, back pain, amputations, tendinitis, fractures, and muscle traumas, as well as age-related degenerative conditions such as osteoporosis. Interventions for the MSD group focus on restoring physical functionality, reducing pain, and enhancing mobility. These include physical rehabilitation and occupational therapy, along with preventative strategies such as exercise regimens and ergonomic adaptations. In severe cases, surgical procedures may be required to address structural damage or improve physical capacity. Pre and post assessments were summarized in Table 1. 

### 3.3. Quality of Life Results

#### 3.3.1. Physical Component of the SF-36

This component evaluates patients’ perception of their physical health, including aspects such as the ability to perform physical activities, role limitations due to physical health problems, bodily pain, and vitality perception. In this study, 388 patients participated in the evaluation of the physical component. Pre-treatment results showed a median score of 28.13 points, with an interquartile range of from 19 to 39 points, indicating that patients perceived considerable limitations in their physical health at the start of the interventions.

After the rehabilitation program, the median score for the physical component significantly increased to 40.31 points, with an interquartile range of from 27 to 54 points. The Wilcoxon test, used to compare pre- and post-treatment scores, showed statistical significance with a *p* < 0.001, confirming that this improvement is significant. The median difference was 10 points, with an interquartile range of 0.3 to 19 points, reflecting a positive impact on patients’ physical functionality.

**Figure 2:**
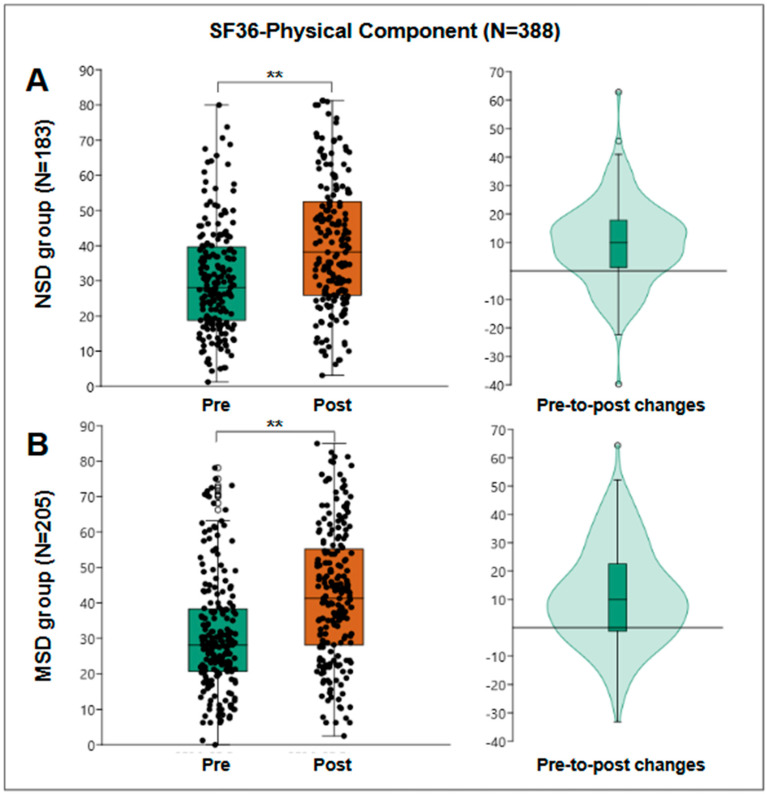
SF36 physical component results. (**A**) The results of the NSD group (N = 138), a box plot of pre- and post-results (**left**), and a violin plot of the individual differences (**right**). (**B**) The results of the MSD group (N = 205), a box plot of pre- and post-results (**left**), and a violin plot of the individual differences (**right**). **: *p* < 0.001.

For the NSD group, 183 patients were evaluated, obtaining a pre-treatment median of 28.13 points and post-treatment median of 38.13 points, obtaining a pre-post median change of 10 points with an interquartile range of from 1.25 to 17.81 and a statistically significant Wilcoxon W = 14,014; r = 0.59; *p* < 0.001 (Figure 2A).

For the MSD group, 205 patients were evaluated, obtaining a pre-treatment median of 28.13 points and post-treatment of 41.25 points, obtaining a median pre-post change of 10 points with an interquartile range of from −1.3 to 22.5 and a statistically significant Wilcoxon W = 17,045; r = 0.56; *p* < 0.001 (Figure 2B).

#### 3.3.2. Mental Component of the SF-36

The mental component of the SF-36 evaluates aspects related to emotional and psychological well-being, such as vitality, social functioning, and overall mental health. In the pre-treatment evaluation, the group of 388 patients had a median score of 48 points for the mental component, with an interquartile range of from 35 to 63 points. After the program, the median score increased to 62.5 points, with an interquartile range of from 44 to 81 points, suggesting a significant improvement in patients’ emotional and psychological state. The Wilcoxon test demonstrated statistical significance with a *p* < 0.001.

**Figure 3:**
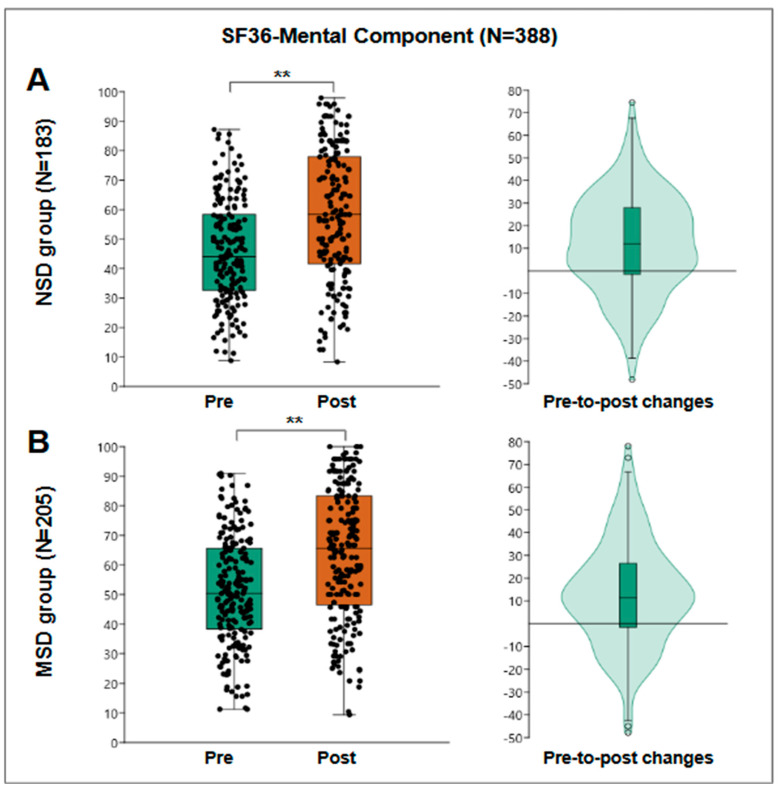
SF36 mental component results. (**A**) The results of the NSD group (N = 138), a box plot of pre- and post-results (**left**), and a violin plot of the individual differences (**right**). (**B**) The results of the MSD group (N = 205), a box plot of pre- and post-results (**left**), and a violin plot of the individual differences (**right**). **: *p* < 0.001.

For the NSD group, 183 patients were evaluated, obtaining a pre-treatment median of 44.1 points and post-treatment median of 58.33 points, obtaining a median pre-post change of 12 points with an interquartile range of from −1.6 to 27.95 and a statistically significant Wilcoxon W = 13,276; r = 0.5; *p* < 0.001 (Figure 3A).

For the MSD group, 205 patients were evaluated, obtaining a pre-treatment median of 50.3 points and post-treatment median of 65.6 points, obtaining a median pre-post change of 11.4 points with an interquartile range of from −1.6 to 26.5 and a statistically significant Wilcoxon W = 16,380; r = 0.49; *p* < 0.001 (Figure 3B).

### 3.4. Independence in Activities of Daily Living

The Barthel Index was evaluated in 504 patients to measure their level of independence in basic activities of daily living. The results showed a pre-treatment median score of 85 points, with an interquartile range of from 70 to 95 points. Subsequently, the median increased to 90 points, with an interquartile range of from 76 to 100 points. The Wilcoxon test indicated a significant improvement in the functional autonomy of the patients (*p* < 0.001).

**Figure 4:**
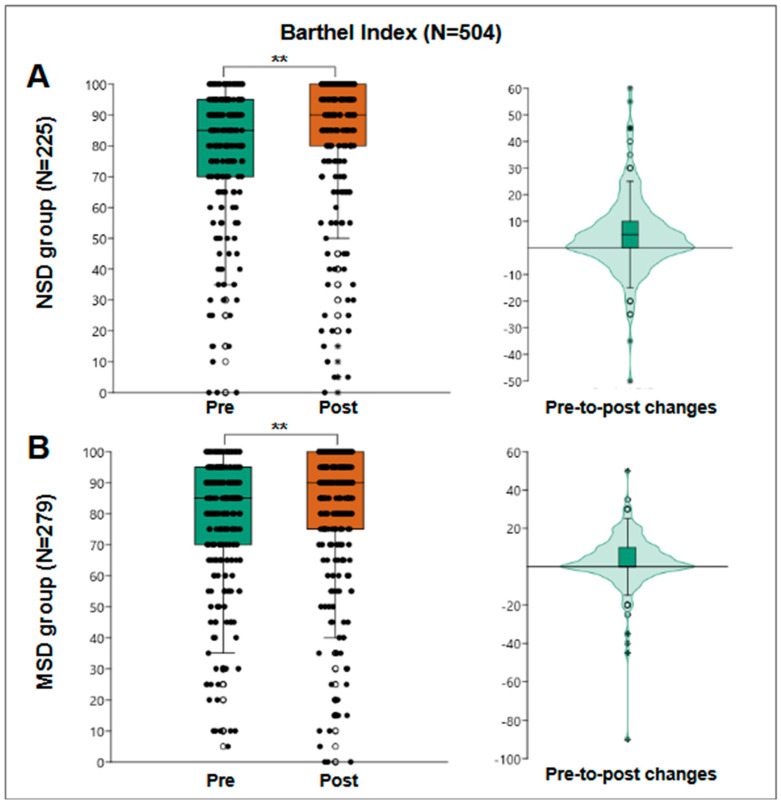
Barthel Index results. (**A**) The results of the NSD group (N = 225), a box plot of pre- and post-results (**left**), and a violin plot of the individual differences (**right**). (**B**) The results of the MSD group (N = 279), a box plot of pre- and post-results (**left**), and a violin plot of the individual differences (**right**). **: *p* < 0.001.

Figure 4A shows the results of the NSD group, where 225 patients were evaluated, obtaining a pre-treatment median of 85 points and post-treatment of 90 points, obtaining a median pre-post change of 5 points with an interquartile range of from 0 to 10 and a statistically significant Wilcoxon W = 10,303; r = 0.38; *p* < 0.001. Within those assessed, 26 users started and finished with the maximum score of 100 points. When reanalyzing the pre-to-post change among the remaining 199 patients, the median remained at 5 points, and the interquartile range changed from −5 to 10 points.

Figure 4B presents the results of 279 patients, all from the MSD group, obtaining a pre-treatment median of 85 points and post-treatment median of 90 points, which determined a median pre-to-post change of 0 points with an interquartile range of from 0 to 10 and a statistically significant Wilcoxon W = 13,515; r = 0.35; *p* < 0.001. Among those assessed, 34 users started and finished with a maximum score of 100 points. Looking again at the pre-to-post change among the remaining 245 patients, the median change was 5 points, with an interquartile range of from −2.5 to 10 points, better reflecting the behavior of the group with no ceiling effect.

### 3.5. Depressive Symptoms

The Beck Depression Inventory was administered to 193 patients to evaluate the presence and severity of depressive symptoms. The pre-treatment median score was 12 points, with an interquartile range of from 7 to 17 points, which decreased to 9 points post-treatment, with an interquartile range of from 6 to 14 points. The Wilcoxon test indicated a significant reduction in depressive symptoms (*p* < 0.001).

**Figure 5:**
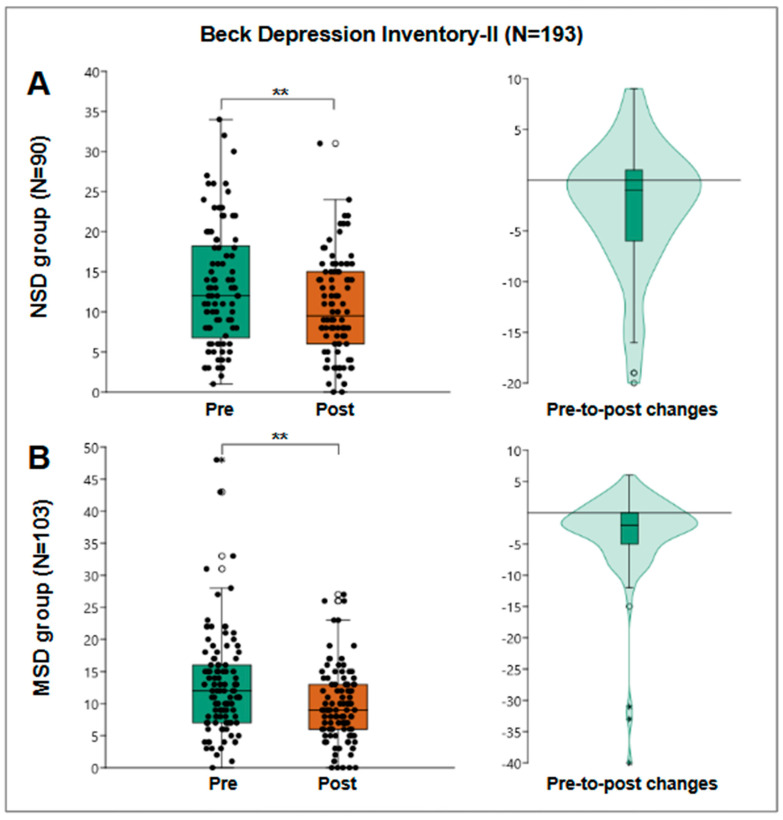
Beck Depression Inventory-II results. (**A**) The results of the NSD group (N = 90), a box plot of pre- and post-results (**left**), and a violin plot of the individual differences (**right**). (**B**) The results of the MSD group (N = 103), a box plot of pre- and post-results (**left**), and a violin plot of the individual differences (**right**). **: *p* < 0.001.

For the NSD group, 90 patients were evaluated, obtaining a pre-treatment median of 12 points and post-treatment median of 9.5 points, obtaining a pre-post median change of −1 points with an interquartile range of from −6 to 1 and a statistically significant Wilcoxon W = 2349; r = 0.39; *p* < 0.001 (Figure 5A).

For the MSD group, 103 patients were evaluated, obtaining a median pre-treatment of 12 points and post-treatment of 9 points, obtaining a median pre-post change of −2 points with an interquartile range of from −5 to 0 and a statistically significant Wilcoxon W = 3799; r = 0.55; *p* < 0.001 (Figure 5B).

### 3.6. Balance and Fall Risk

The Berg Balance Scale, evaluated in 487 patients, showed a pre-treatment median score of 41 points, with an interquartile range of from 28 to 49 points, and a post-treatment median score of 47 points, with an interquartile range of from 34 to 54 points. The Wilcoxon test indicated a significant improvement in balance and a reduction in fall risk (*p* < 0.001).

**Figure 6:**
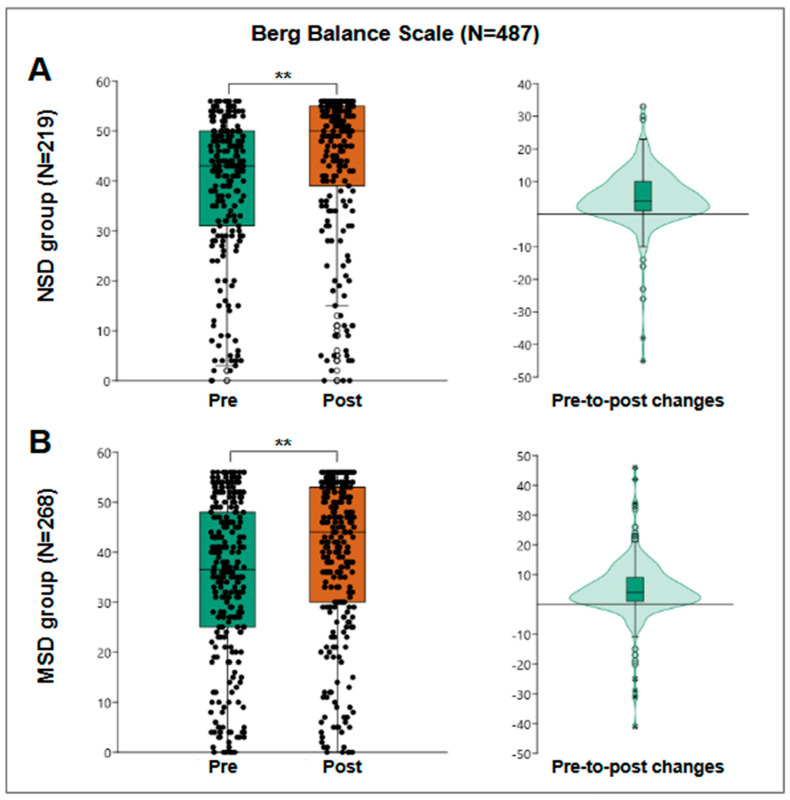
Berg Balance Scale results. (**A**) The results of the NSD group (N = 90), a box plot of pre- and post-results (**left**), and a violin plot of the individual differences (**right**). (**B**) The results of the MSD group (N = 103), a box plot of pre- and post-results (**left**), and a violin plot of the individual differences (**right**). **: *p* < 0.001.

For the NSD group, 219 patients were evaluated, obtaining a pre-treatment median of 43 points and post-treatment of 50 points (Figure 6A), obtaining a median pre-post change of 4 points with an interquartile range of from 1 to 10 and a statistically significant Wilcoxon W = 17,340; r = 0.63; *p* < 0.001. However, within those assessed, eight users started and finished with the maximum score of 56 points. Looking again at pre-to-post change among the remaining 211 patients, the median change was 5 points, with an interquartile range of from 1 to 10 points, better reflecting the behavior of the group.

For the MSD group, 268 patients were evaluated, obtaining a median pre-treatment score of 36.5 points and a post-treatment score of 44 points (Figure 6B), obtaining a median pre-post change of 4 points with an interquartile range of from 1 to 9 and a statistically significant Wilcoxon W = 25,751; r = 0.6; *p* < 0.001. However, within those assessed, nine users started and finished with the maximum score of 56 points. When reanalyzing the pre-to-post change among the remaining 268 patients, both the median and IQ range remained the same.

## 4. Discussion

This study evaluated the impact of a comprehensive, multidisciplinary rehabilitation program on various health and well-being outcomes in older adults and individuals with neuromusculoskeletal disabilities. The program employed a personalized approach, tailoring interventions to individual needs and addressing the physical, emotional, and social dimensions of rehabilitation.

A key finding was the significant improvement in motor function, particularly balance. Participants exhibited a notable increase in their Berg Balance Scale scores, rising from an average of from 39 to 47 points. This improvement reflects a reduced risk of falls and enhanced functional stability, which are critical aspects of rehabilitation for this population [33]. The positive change was consistent across both neurological and musculoskeletal subgroups, with the neurological group improving from 43 to 50 points and the musculoskeletal group from 36.5 to 44 points. These findings underscore the program’s effectiveness across diverse conditions and align with existing evidence highlighting the role of targeted exercise programs in enhancing balance and reducing fall risk [34]. Specifically, the inclusion of exercises designed to challenge and improve postural control, as proposed by Horak et al. (2009) and their BESTest assessment tool, likely contributed significantly to these observed outcomes [35]. By leveraging such tools to identify and address specific balance deficits, rehabilitation programs can be optimized to maximize individual progress and functional gains.

Beyond physical improvements, this study revealed a moderate yet meaningful increase in functional independence, as measured by the Barthel Index. Participants’ scores increased from a median of from 85 to 90 points, indicating a greater capacity to perform activities of daily living. This gain in independence is crucial for enhancing quality of life, not only for the individuals themselves but also for their caregivers, who often shoulder substantial responsibilities in daily assistance [36]. The use of standardized assessments like the Barthel Index is particularly valuable, as it enables healthcare professionals to objectively monitor progress, evaluate intervention efficacy, and identify areas requiring further attention [37]. It is important, however, to take into account the pre- and post-treatment ceiling effect of this test in order to correctly reflect the potential changes in each individual. This approach supports the development of highly individualized rehabilitation plans that cater to specific needs and promote long-term well-being.

Importantly, this study also addressed participants’ emotional well-being. A significant reduction in depressive symptoms was observed, with Beck Depression Inventory scores decreasing from a median of from 12 to 9 points in the musculoskeletal group and from 12 to 9.5 in the neurological group. This finding underscores the interplay between physical and mental health, supporting growing evidence on the antidepressant effects of exercise and physical activity [38,39,40]. Regular physical activity has been shown to improve mood, alleviate feelings of hopelessness, and enhance motivation, all of which are vital for active engagement in the rehabilitation process and successful recovery. However, this study recognizes that some participants may require additional support to address persistent depressive symptoms, highlighting the importance of incorporating psychological interventions, such as cognitive–behavioral therapy, into comprehensive rehabilitation programs.

Additionally, this study explored the broader impact of the program on participants’ quality of life using the SF-36 questionnaire. Improvements were observed in both physical and mental component scores. The physical component, which encompasses aspects such as physical functioning, bodily pain, and general health perceptions, showed a substantial increase, with the musculoskeletal group improving from 28.13 to 41.25 points and the neurological group from 28.13 to 38.13 points. These results suggest that the program positively influenced participants’ perceptions of their physical health and vitality, contributing to an enhanced sense of well-being [15]. Similarly, the mental component, which assesses factors like vitality, social functioning, and emotional well-being, also improved, rising from 47.5 to 62.5 points. This indicates that the program not only enhanced physical functioning but also had a positive impact on participants’ mental and emotional states, fostering resilience and motivation [41]. These findings reinforce the importance of adopting a holistic approach to rehabilitation, addressing both physical and mental health to achieve comprehensive and enduring recovery.

Despite these promising results, this study acknowledges certain limitations. The absence of a control group hinders the ability to definitively attribute the observed improvements solely to the interventions provided. Variability in treatment duration and the number of sessions among participants could also act as confounding factors, as adherence to rehabilitation programs is critical for achieving optimal outcomes. As highlighted by McLean et al. (2010), integrating motivational and cognitive–behavioral strategies can be instrumental in enhancing patient engagement and improving short-term outcomes [42]. Furthermore, the reliance on self-reported measures, while valuable for capturing subjective experiences, may introduce biases such as social desirability. Deshpande et al. (2011) emphasize the need to complement self-reported data with objective measures to ensure a more robust and unbiased evaluation [43]. Future research should address these limitations by incorporating a control group, standardizing treatment protocols, and combining objective and subjective assessments to provide a more comprehensive and rigorous evaluation of intervention efficacy.

Nevertheless, this study offers valuable insights for clinical practice in home-based settings. It highlights the importance of a holistic, patient-centered approach to rehabilitation, where interventions are tailored to individual needs and address physical, emotional, and social dimensions. The evidence of significant improvements across multiple domains underscores the effectiveness of such an approach in meeting the complex needs of older adults and individuals with disabilities [41,44].

Furthermore, the findings suggest that the intensity and duration of interventions are critical for optimizing therapeutic outcomes. The substantial improvements observed in this study, with participants receiving a median of 135 sessions over 16 months, indicate that higher-intensity and longer-duration programs may yield more comprehensive and lasting benefits compared to shorter interventions. This aligns with the findings of Barría et al. (2024), which implemented a shorter program and reported significant improvements in balance but limited progress in emotional or functional domains [45]. These results emphasize the need for rehabilitation professionals to carefully consider intervention dosages, adjusting the frequency and intensity of sessions to align with individual needs and goals [10,11]. Ongoing monitoring and assessment throughout the rehabilitation process, including regular mental health evaluations and psychological support, are crucial to ensuring sustained progress and maximizing long-term benefits.

Overall, this study underscores the value of a personalized, intensive rehabilitation approach that integrates physical, emotional, and social components to promote comprehensive recovery in populations with complex needs. Future research should prioritize long-term follow-up studies to evaluate the durability of these improvements and explore strategies to sustain the benefits of rehabilitation interventions over time [46].

## 5. Conclusions

In conclusion, this study provides significant evidence that structured rehabilitation interventions can improve motor function, functional independence, and emotional well-being in individuals with neuromusculoskeletal conditions. The results demonstrate that participants experienced notable improvements in balance and a reduction in fall risk, as well as an increase in their perception of physical and mental health.

This study reinforces the relevance of personalized and intensive care in rehabilitation and underscores the importance of a multidisciplinary approach that considers both the physical and emotional aspects of recovery. Future studies should focus on evaluating the long-term effects of these interventions and exploring additional measures for a more comprehensive assessment of improvements in motor function and quality of life.

## Figures and Tables

**Table 1:** Results of clinical outcomes expressed as median points for pre- and post-intervention assessments, separated by diagnosis group. *p* value and r were obtained from Wilcoxon paired test.

Clinical Outcome	Nervous System Group					Musculoskeletal System Group				
	N	Pre	Post	Effect Size (r)	*p* Value	N	Pre	Post	Effect Size (r)	*p* Value
SF36-PC	183	28.1	38.1	0.59	<0.001	205	28.1	41.3	0.56	<0.001
SF36-MC	183	44.1	58.3	0.5	<0.001	205	50.3	65.6	0.49	<0.001
Barthel	225	85	90	0.38	<0.001	279	85	90	0.35	<0.001
Beck	90	12	9.5	0.39	<0.001	103	12	9	0.55	<0.001
Berg	219	43	50	0.63	<0.001	268	36.5	44	0.6	<0.001

## Data Availability

The data are available upon reasonable request. To access them, please contact PB by email at pbarria@rehabilitamos.org.
